# Bisphosphonate-related Femoral Shaft Fracture

**DOI:** 10.5811/cpcem.2019.10.45007

**Published:** 2019-12-17

**Authors:** Jesse Kellar, Alan Givertz, Jessica Mathias, Jessica Cohen

**Affiliations:** Saint Agnes Medical Center, Department of Emergency Medicine, Fresno, California

## Abstract

The efficacy of using bisphosphonate therapy to treat osteoporotic patients is becoming more widely known, but the potential side effects may not be. While this class of drugs is generally safe, concerns have emerged regarding risks of atypical subtrochanteric fractures associated with long-term use. There have been a number of case reports discussing the association of patients on bisphosphonates who suffer a non-traumatic or a low-energy mechanism of injury atypical of subtrochanteric fractures. The purpose of this case report is to raise awareness of this potential side effect and provide increased clinical suspicion for this rare type of fracture.

## INTRODUCTION

The risk of fracture in osteoporotic patients is high due to the low bone-mineral density and poor bone quality, which develops as an individual ages. It has been estimated that more than 200 million people worldwide have osteoporosis, including about 75 million in the United States, Europe, and Japan.^1^ The condition is especially common in postmenopausal women as approximately half of all women over the age of 50 will experience an osteoporosis-related fracture at some point during their lifetime.^2^ Twenty percent of these patients will die within 12 months after sustaining a fracture.^2^

The National Institutes for Health and Care Excellence recommends bisphosphonates as the treatment of choice for all patients over the age of 50 who are at risk of a fragility fracture, and for those patients under age 50 with a prior history of fragility fracture. The most commonly prescribed drugs from the bisphosphonate class are alendronate, risedronate, ibandronate, and zoledronic acid. These function to inhibit bone resorption and interfere with the action of bone-resorbing osteoclasts.^3^ Bisphosphonates can be administered orally or intravenously in a wide range of doses. While bisphosphonates have proven to reduce the occurrence of spinal and hip fractures, long-term users are found to have a higher susceptibility to atypical subtrochanteric fractures.

## CASE REPORT

**Image 1:**
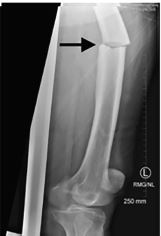
Lateral view of left femur reveals acute mid-femoral shaft fracture with characteristic lateral cortex beaking consistent with bisphosphonate fracture (arrow).

**Image 2:**
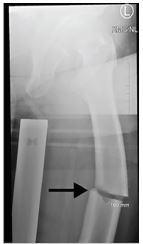
Frontal view of the pelvis reveals acute mid-femoral shaft fracture with characteristic lateral cortex beaking consistent with bisphosphonate fracture (arrow).

A 79-year-old female with significant past medical history including osteoporosis presented to the emergency department (ED) with severe, throbbing left hip pain after a ground-level fall. She was walking around a corner when she ran into a doorknob with her left leg. The patient subsequently fell to the ground and was unable to get back up. On the initial paramedic assessment, as well as in the ED, the patient’s left hip was rotated with obvious shortening and major deformity at the left mid femur. The left foot was neurovascularly intact. A radiograph of the femur revealed an acute mid femoral-shaft fracture with characteristic lateral cortex breaking consistent with biphosphonate fracture (Images 1 and 2).

Her prescription history confirmed that she was on aspirin and a 70 milligram tablet of alendronate every seven days from September 2018 through June 2019. She had an orthopedics consultation and underwent a left hip cephalomedullary nail procedure.

## DISCUSSION

In 2005, a report of nine patients on long-term alendronate documented that they had suffered low-energy nonvertebral fractures, with three of the nine patients having an atypical subtrochanteric fracture.^3^ Low-energy fractures are caused by minimal trauma such as bumping into a door, stepping off a curb, or walking. As described in the case study presented above, the patient suffered a ground-level fall after walking into a doorknob. While this patient had immediate severe pain and an obvious deformity after the fall, some patients can have early pain in the affected region weeks to months before sustaining a spontaneous or low-trauma injury.^3^ Of patients who have an atypical subtrochanteric fracture, 32–76% present with persistent non-traumatic pain in the groin or hip.^4^ The pain is most commonly presented in the anterior or lateral thigh or in the groin.

If a patient is on long-term bisphosphonates and presents with this pain, it should be a signal to obtain a radiograph of the lower extremity. While the fracture pathogenesis is still unknown, there is evidence suggesting that long-term bisphosphonate use may stop bone metabolism, limiting the repair of microdamage and creating the risk of low-energy fractures.^5^ Radiography is the first step to rule out this type of fracture. An anteroposterior and lateral plain radiograph of the hip, including the full diaphysis of the femur, should be performed.^2^ If images appear normal but clinical suspicion remains high, a technetium bone scan or magnetic resonance imaging (MRI) may be performed. In order to confirm the diagnosis of an atypical subtrochanteric fracture, MRI is required even if initial findings were discovered on a radiograph. Once an atypical fracture is confirmed, the bisphosphonate regimen must be stopped and patients should be administered daily calcium and vitamin D supplementation.

**CPC-EM Capsule**

What do we already know about this clinical entity?

Bisphosphonate therapy is commonly used to treat osteoporosis, and help prevent bone loss and future fractures. A potential side effect is atypical femoral fractures.

What makes this presentation of disease reportable?

This case highlights the need for high clinical suspicion of fracture in patients on bisphosphonate therapy, even those with low mechanism of injury.

What is the major learning point?

Understanding the potential side effects of bisphosphonate therapy, including atypical femoral fractures.

How might this improve emergency medicine practice?

This case will help elevate the suspicion for a fracture in patients on bisphosphonate therapy who come into the emergency department after suffering from a low mechanism of injury.

## CONCLUSION

The benefits versus risks of patients using bisphosphonates should ultimately be decided by the patient’s primary care provider. The purpose of this case report is to raise provider awareness regarding these risks and to have increased clinical suspicion for an atypical subtrochanteric fracture if a patient presents with these signs and symptoms.
